# Inactivation of DltA Modulates Virulence Factor Expression in *Streptococcus pyogenes*

**DOI:** 10.1371/journal.pone.0005366

**Published:** 2009-04-29

**Authors:** Kathleen H. Cox, Eduardo Ruiz-Bustos, Harry S. Courtney, James B. Dale, Morgan A. Pence, Victor Nizet, Ramy K. Aziz, Ivan Gerling, Susan M. Price, David L. Hasty

**Affiliations:** 1 Department of Anatomy and Neurobiology, University of Tennessee Health Science Center, Memphis, Tennessee, United States of America; 2 Department of Medicine, University of Tennessee Health Science Center, Memphis, Tennessee, United States of America; 3 Department of Veterans Affairs Medical Center, Memphis, Tennessee, United States of America; 4 Department of Molecular Sciences, University of Tennessee Health Science Center, Memphis, Tennessee, United States of America; 5 Department of Pediatrics, University of California San Diego, La Jolla, California, United States of America; 6 Department of Microbiology and Immunology, Faculty of Pharmacy, Cairo University, Cairo, Egypt; Theodor-Boveri-Institut fur Biowissenschaften, Wurzburg, Germany

## Abstract

**Background:**

D-alanylated lipoteichoic acid is a virtually ubiquitous component of Gram-positive cell walls. Mutations in the *dltABCD* operon of numerous species exhibit pleiotropic effects, including reduced virulence, which has been attributed to increased binding of cationic antimicrobial peptides to the more negatively charged cell surface. In this study, we have further investigated the effects that mutating *dltA* has on virulence factor expression in *Streptococcus pyogenes*.

**Methodology/Principal Findings:**

Isogenic Δ*dltA* mutants had previously been created in two distinct M1T1 isolates of *S. pyogenes*. Immunoblots, flow cytometry, and immunofluorescence were used to quantitate M protein levels in these strains, as well as to assess their ability to bind complement. Bacteria were tested for their ability to interact with human PMN and to grow in whole human blood. Message levels for *emm*, *sic*, and various regulatory elements were assessed by quantitative RT-PCR. Cell walls of Δ*dltA* mutants contained much less M protein than cell walls of parent strains and this correlated with reduced levels of *emm* transcripts, increased deposition of complement, increased association of bacteria with polymorphonuclear leukocytes, and reduced bacterial growth in whole human blood. Transcription of at least one other gene of the *mga* regulon, *sic*, which encodes a protein that inactivates antimicrobial peptides, was also dramatically reduced in Δ*dltA* mutants. Concomitantly, *ccpA* and *rofA* were unaffected, while *rgg* and *arcA* were up-regulated.

**Conclusions/Significance:**

This study has identified a novel mechanism for the reduced virulence of *dltA* mutants of *Streptococcus pyogenes* in which gene regulatory networks somehow sense and respond to the loss of DltA and lack of D-alanine esterification of lipoteichoic acid. The mechanism remains to be determined, but the data indicate that the status of D-alanine-lipoteichoic acid can significantly influence the expression of at least some streptococcal virulence factors and provide further impetus to targeting the *dlt* operon of Gram-positive pathogens in the search for novel antimicrobial compounds.

## Introduction

Lipoteichoic acids (LTAs) are abundant surface components of virtually all Gram-positive bacteria, the structural details of which vary somewhat among the different species [1]. *Streptococcus pyogenes* LTA is composed of a highly charged polyglycerolphosphate (PGP) moiety extending into the cell wall and covalently linked to a diglucosyl-diacylglycerol inserted in the outer leaflet of the cell membrane. Protonated D-alanine, the only substituent of the PGP chains of *S. pyogenes*, is ester-linked to the C-2 of roughly half of the glycerol phosphate units. These positively charged substitutions provide counterions that determine the net anionic charge of LTA. Although it has been shown that LTA is essential for Gram-positive bacterial growth and cell division [2], its precise functions at the bacterial surface are not known. It has been hypothesized that LTA contributes to metal cation homeostasis, assists the “trafficking” of ions, nutrients, proteins and antibiotics, modulates the activity of autolysins and defines the electrochemical properties of the cell wall [1]. The D-alanylation of LTA is accomplished by proteins encoded by the four genes of the *dlt* operon, *dltABCD* [3], [4]. The level of D-alanylation is not only affected by *dlt* gene activity, but also by pH, since the ester bond is labile at and above neutrality [5]. It has been shown in several Gram-positive pathogens that failure of the D-alanylation system results in bacteria with significantly reduced virulence properties [4], [6]–[16].

A major correlate of virulence of *S. pyogenes* is their ability to resist phagocytosis in the non-immune human host [17]. M protein is an alpha-helical, coiled-coil protein that extends from the surface of *S. pyogenes* with its hypervariable NH_2_-terminus exposed to the external environment and its conserved COOH-terminus attached to cell wall peptidoglycan by an LPXTG anchoring motif common to many Gram-positive surface proteins [18]. The gene for M protein, *emm*, is located in a regulon whose primary known control is through the upstream positive regulator, *mga* [19]–[22]. Inactivation of the *emm* gene can result in an avirulent organism that can no longer resist phagocytosis [23]. Fibrinogen binding to M proteins confers resistance to phagocytosis by preventing the deposition of complement on the bacteria [24]–[26].

Previous studies of several bacterial species, including *S. pyogenes*, have concluded that an increase in net negative surface charge of *dltA* mutants is most likely responsible for many of the resulting phenotypes, including decreased adherence to epithelial cells [6], [13] and increased sensitivity to cationic antimicrobial peptides (AMPs; [4], [13], [27]). The data reported here reveal a novel effect of the inactivation of *dltA* and suggest another mechanism that contributes to the overall level of virulence of Δ*dltA* mutants of *S. pyogenes*.

## Materials and Methods

### Bacterial strains and mutagenesis strategies

The two WT isolates of *S. pyogenes* M1T1 utilized in these studies (*S. pyogenes* 5448 and *S. pyogenes* 8004) were described previously [28]. Bacteria were grown in Todd-Hewitt broth with 1% yeast extract (THY) unless indicated otherwise. In-frame *dltA* mutations in 5448 and 8004 were created by two different procedures. 5448Δ*dltA* was constructed by replacing the *dltA* gene with the chloramphenicol acetyltransferase gene (*cat*) [13], [29]. Lack of D-alanylation in 5448Δ*dltA* was previously shown by extraction of D-ala from parent and mutant strains by mild alkaline hydrolysis and quantification by HPLC (29). 8004Δ*dltA* was created independently and by a different procedure [30] using the temperature-sensitive *E. coli*-streptococcus shuttle vector, pG+Host9 plasmid [31]. Following the procedures outlined by Maguin and co-workers, nt 470–1062 were deleted in-frame from *dltA* [30]. Mutations were confirmed by sequencing. Lack of D-alanylation in 8004Δ*dltA* was previously shown by amino acid and NMR analysis of purified LTA (30). Growth of either of the mutants in THY is indistinguishable from that of the parent, at least through early stationary phase. 5448Δ*dltA* was complemented using plasmid p*dltA*, as described [13], creating 5448Δ*dltA*(p*dltA*). In p*dltA*, transcription is under the control of the erythromycin promoter. We have never been able to successfully transform 8004Δ*dltA* with the p*dltA* plasmid, so complementation studies could not be conducted. We presume that this was due to hyperencapsulation or another unidentified consequence of the Δ*dltA* mutation in the CovS- background strain. However, it is informative that in almost every other way 8004Δ*dltA* mimicked 5448Δ*dltA*, suggesting that the observed phenotypes were due to the mutation in *dltA*.

### Assays for resistance to phagocytosis

The ability of parent and mutant strains to interact with phagocytic cells and to grow in human blood was tested using two different assays, respectively. For opsonophagocytosis tests, the bacteria were grown to an OD_600_ of 0.1, and 25 µl aliquots were added to 125 µl PBS and 350 µl heparinized, non-immune human blood. Standard blood smears were done at 0, 5, 10, 20 and 40 min and stained with Wright's stain. The number of bacterial chains and the number of bacteria/chain scored either as being associated with PMNs or not. At least 750 bacterial chains were counted for each point. Because results are expressed in percent of bacteria in PMN, standard errors would not be valid.

To test their ability to grow in human blood, bacteria were grown to an OD_600_ of 0.08, diluted 1∶10,000 in THY and 50 µl aliquots were added to tubes containing 0.45 ml of heparinized, non-immune human blood. Two different donors were used and tests were done on different days. Mixtures were rotated for 2 or 3 h at 37°C and the number of CFU was determined before and after incubation by plating dilutions on blood agar plates. The three separate assays performed are reported, because of the inherent variability associated with these assays.

### Quantitative RT-PCR (qRT-PCR)

RNA was isolated at mid-log phase using the RNeasy Mini Isolation Kit with the RNAprotect Bacteria Reagent (Qiagen) and was reverse-transcribed into cDNA. One µg total bacterial RNA was used as the template to synthesize cDNA. Duplicate assays with 50 ng cDNA were run for each gene tested. qRT-PCR was performed using an ABI Prism 7900 instrument. Expression levels of test genes were normalized to expression levels of gyrase (*gyr*) message. Primers utilized are given in Table 1. For *emm* and *mga*, results were confirmed using two different primer sets. Expression is given as the relative message level in mutant strains compared to the WT message levels, which were set at 1.

**Table 1:** Oligonucleotide primers used for qRT-PCR.

Gene	Primer	Sequence (5′–3′)
*gyrA*	GyrA-F	GAA CGC CAA AGC CAA GCT AT
	GyrA-R	TTG AAT CTT ATC ACG TTC CAA ACC
*emm1*	Emm1A-F	CTC CAG CTG TTG CCA TAA CAG T
	Emm1A-R	AGA CAG TTA CCA TCA ACA GGT GAA AC
	Emm1B-F	TGA TTC CTT TGC TAA GTC ATA GTC TTG
	Emm1B-R	TGG AAG TTG CAG GAA GAG ATT TT
*sic*	SIC-F	GCC AGC TGA AAA CCC TCT ATC A
	SIC-R	CCT CGT GTG CCA GAA AAA CC
*mga*	MgaA-F	TGA AGG ATG ACA GCT ACT TCG TAT TT
	MgaA-R	TGG GTT CAT CTC CTT GAT GAG TT
	MgaB-F	CAA GGA GAT GAA CCC AGT TGG T
	MgaB-R	ATC GGT TAT GCG TTT GAT AGC A
*ccpA*	CcpA-F	TGA ATA CAG ATG ATC CCC TTA CAA TTT
	CcpA-R	CGA CTA ACG GTT GCC ATT GA
*rof*	Rof-F	ATT CCG ATA ACC AAG GAG AGG TT
	Rpf-R	GAC GCA GCA AGA CAA AAA CAC T
*rgg*	Rgg-F	ATT ATT GGC CTC ATA AAT GGT AAA GAG
	Rgg-R	TGA TGG ATC GTT TTG CAA TTA AGT
*arcA*	ArcA-F	ACG GTT ACG GGT TTC TGA GAA C
	ArcA-R	TTT ATT TCA CAC GGG ACC CAT T

a F and R indicate forward and reverse primers in each case.

b Two different primer sets were used for emm1 and mga. Essentially identical results were obtained from both.

### Flow cytometry and immunolocalization

Streptococci were grown to mid-log phase and 0.5 ml of washed bacteria were added to either 0.5 ml of fresh, heparinized plasma from non-immune donors or to 0.1%BSA in PBS as a control, incubated for 10 min at 37°C and washed. Bacteria were fixed in formalin, blocked in 0.1% BSA and washed. For flow cytometry and IF analysis of C3 deposition, bacteria were then incubated with a 1∶500 dilution of FITC-conjugated goat anti-human C3 IgG (MP Biomedicals, Irvine, CA). For IF analysis of M1 protein, the bacteria were incubated in a 1∶200 dilution of a rabbit polyclonal antibody against the pepsin-digested fragment of M1 [32] followed by incubation with Alexa Fluor 594-conjugated donkey anti-rabbit IgG secondary antibody (Molecular Probes). To visualize nuclei, DAPI was included in the mounting media. For flow cytometry of both C3 and M1 levels, incubation with the M1 primary and secondary antibody preceded the incubation with the FITC-conjugated anti-human C3 probe. The secondary antibody for the M1 analysis was a donkey anti-rabbit Fab' Cy5. IF images were acquired using AxioVision 3.0 (Zeiss) software and a Zeiss Axioplan 2 microscope. Flow cytometry was carried out using the FACSCalibur flow cytometer (Becton, Dickinson and Company, Franklin Lakes, NJ) and Cell Quest Pro software.

### Protein extraction, Western blotting and 2-D PAGE

Cell wall extracts and supernatants were prepared from bacteria grown to mid-log phase according to the hot acid procedure of Lancefield [33] and also by the mutanolysin/lysozyme procedure of Biswas et al. [34] to ensure that the results were not simply due to differences in extractability of M protein from the WT and mutant strains. The cells were centrifuged and the supernatant proteins were isolated by 10% TCA precipitation and acetone wash, followed by resuspension in sample buffer. For 1-dimensional analysis and immunoblots, equal amounts of protein were added to each gel lane. Total proteins were analyzed by SDS-PAGE followed by silver staining. For Western blots, proteins on nitrocellulose membranes were incubated with anti-PepM protein described previously [32], followed by incubation with goat anti-rabbit IgG peroxidase-conjugate (ICN/Cappel). Immunoreactive species were detected by ECL Western blotting detection reagents (Amersham Biosciences). For 2-dimensional gels, proteins were separated using a pI range 4–7 strip in the first dimension and a 12% SDS-PAGE homogeneous gel in the second dimension. Gels were stained with SYPRO ruby stain.

## Results

### Bacterial strain comparison

*S. pyogenes* strains 5448 and 8004 were isolated from severe invasive infections [28]. They are highly related strains that contain identical *emm1.0* gene sequences and the same pyrogenic exotoxin (Spe) genotype and random amplified polymorphic DNA (RAPD) profile [35]. They differ, however, in certain respects, including their Spe phenotype. *S. pyogenes* 5448 is SpeB+/SpeA−, while *S. pyogenes* 8004 is SpeB−/SpeA+ [35]. This shift in SpeA and SpeB expression levels is a hallmark reflection of *covS* mutations characteristic of many invasive M1T1 isolates and is reproduced upon experimental animal passage of M1T1 isolates [36], [37]. *S. pyogenes* 8004 bears a frameshift mutation in *covS* caused by a ‘gaaa’ deletion that disrupts a functional domain of CovS in a manner similar to the inactivating mutation found in 5448AP [37]. In both 5448Δ*dltA* and 8004Δ*dltA*, lack of LTA alanylation resulted in increased sensitivity to low pH and AMPs, as well as to decreased adhesion to epithelial cells in *S. pyogenes* [13] (Cox and Hasty, unpublished data). The parent and mutant strains do not exhibit any notable differences in their growth curves in THY through early stationary phase. All of the experiments reported here were done on organisms in the log phase of growth.

### M Protein in Extracts

**Figure 1:**
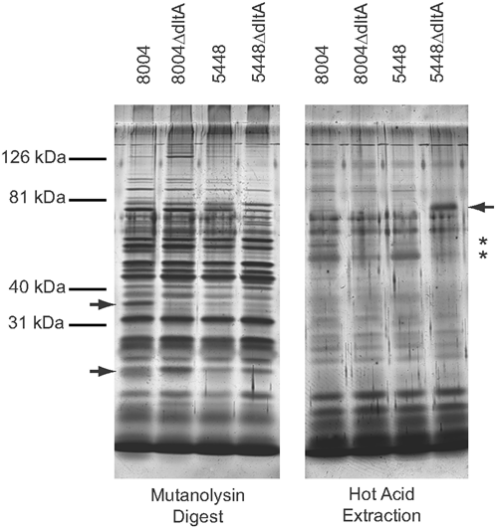
Cell wall protein extracts of parent and Δ*dltA* mutants. Bacteria were grown to mid-log phase and subjected either to lysozyme-mutanolysin digestion or hot acid extraction to obtain cell wall proteins. The material obtained was analyzed by SDS-PAGE followed by silver staining. Asterisks indicate positions where major M protein bands would migrate. Arrows indicate other proteins whose expression also appears to be altered in mutant compared to WT strains.

**Figure 2:**
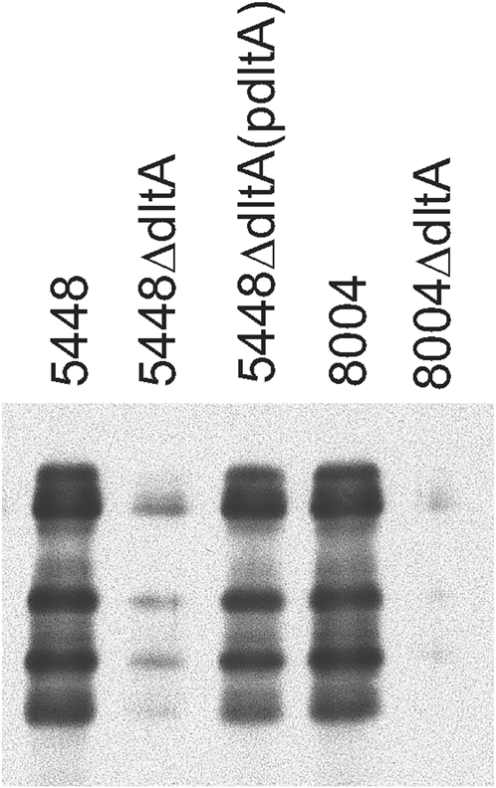
Immunoblot of cell wall extract with anti-M1 protein antibody. Bacteria were grown to mid-log phase and their cell wall proteins were extracted with hot acid. The extracts were loaded exactly as in Fig. 1, which shows that total protein in each lane was virtually identical. After separating proteins by SDS-PAGE and blotting to nitrocellulose, the M protein containing bands were localized with an anti-M1 antibody.

It was previously reported that levels of M protein were similar in *S. pyogenes* 5448 and the 5448Δ*dltA* mutant [13]. Because mutating *dltA* has been shown to alter the set of surface associated proteins of *S. aureus* and *S. gordonii* [9], [38], we were interested in asking whether some other surface proteins of *S. pyogenes* might be affected by mutating *dltA*. Thus, we examined cell wall proteins released by enzymatic treatment (lysozyme+mutanolysin) or by hot acid, a classic protocol for extraction of M protein [33]. There were several obvious quantitative and qualitative differences in the silver-stained proteins released from WT and mutant strains by both procedures (Fig. 1). We were surprised to find that probing blots of extracts with antibody against M1 protein indicated that the amount of M1 protein isolated from both of the Δ*dltA* mutants using hot acid was significantly less than that isolated from either of the WT strains (Fig. 2). The differences in M1 protein were also observed in mutanolysin/lysozyme extracts, as well as when bacteria were extracted using phage lysin, PlyC [39] (data not shown). The M1 protein deficit in 5448Δ*dltA* was largely reversed by complementation with p*dltA* (Fig. 2), suggesting that the reduction of cell wall M1 protein in the mutant was primarily attributable to the loss of *dltA* and not to a secondary mutation. It has not yet been possible to test complementation in 8004Δ*dltA*, as indicated, due to difficulties in transforming the strain with the p*dltA* plasmid. The multiple protein bands reacting with antibody against M1 protein are typical of hot acid extracts and were observed for all of the WT and recombinant strains. The specific acid cleavage sites are not presently known, but the largest of the immunoreactive species co-migrates with the M1 protein identified in enzymatic digests and also reacts with antibody against a synthetic amino terminal M1 protein peptide (data not shown).

**Figure 3:**
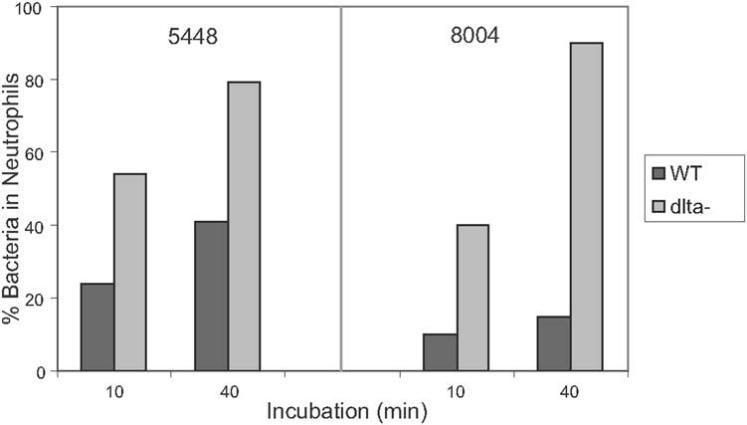
Opsonophagocytosis test. WT and Δ*dltA* mutant bacteria were grown to mid-log phase, washed and incubated in whole human blood for 10 or 40 minutes. Blood samples were placed on slides and stained. The percent of bacteria associated with neutrophils was determined by light microscope analysis.

**Figure 4:**
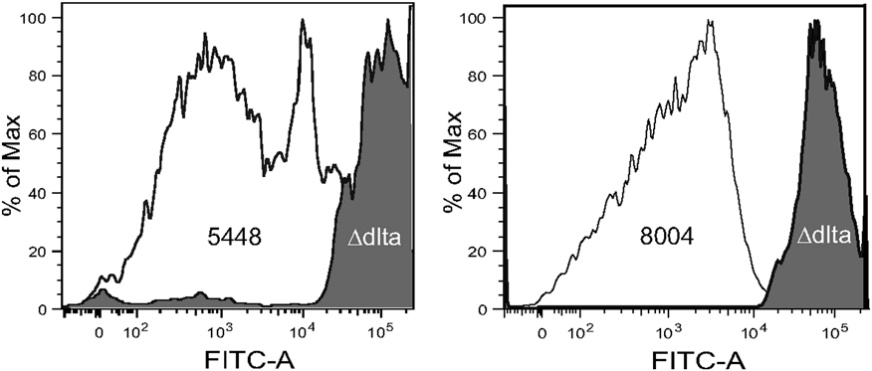
Relative levels of C3 deposition on WT and Δ*dltA* mutant bacteria. WT bacteria (open histogram) and Δ*dltA* mutants (filled histogram) were stained with a fluorescein-labeled antibody against human C3 and analyzed by flow cytometry.

**Figure 5:**
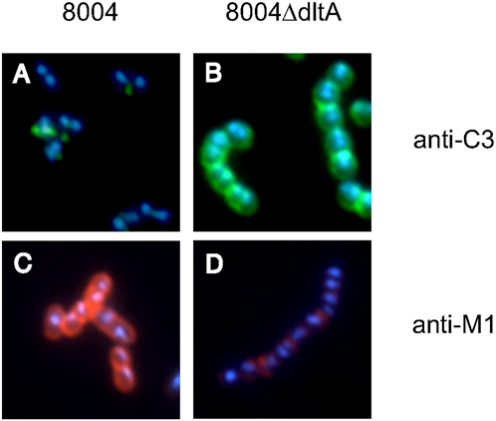
Localization of C3 and M1 by immunofluorescence. WT 8004 cells (Panels A and C) and 8004Δ*dltA* cells (Panels B and D) were stained either with human anti-C3 (Panels A and B) or anti-M1 (Panels C and D) antibodies. DAPI staining (blue) was used to visualize nuclei. Note that the fluorescence associated with M proteins (red) decreases in the *dltA* mutant and the fluorescence associated with C3 dramatically increases (green).

Since surface expression of M protein is critical for *S. pyogenes* to resist phagocytosis, we tested the WT/mutant pairs in opsonization assays. Many fewer WT bacteria were associated with PMNs than either Δ*dltA* mutant at both time points tested (Fig. 3). M protein aids the bacteria by binding fibrinogen and other host proteins, thus inhibiting complement deposition [23]–[26]. Therefore, we examined C3 deposition by flow cytometry to determine its correlation with M1 protein levels. Following incubation of streptococci with human plasma, C3 was quantified using a fluorescein-labeled antibody against human C3. The binding of C3 by the WT differed significantly from that of the Δ*dltA* mutants (Fig. 4). Binding of C3 to both Δ*dltA* mutants was up to 100-fold higher than C3 binding to the parent strains. We also examined C3 deposition by immunofluorescence (IF) (Fig. 5). As was predicted by flow cytometry, chains of the *dltA* mutant showed very similar levels of C3 signal within chains and from chain to chain (Fig. 5 B). The entire cell surface appeared to be labeled, although some chains showed slightly higher label at regions of nascent cell walls. Most WT chains showed no C3 signal (Fig. 5 A), unless the exposure time was extended significantly. However, C3 signal was occasionally detected (Fig. 5A), and in these cases it was mainly confined to forming and newly formed cell walls. The amount and localization of M1 protein on these strains was also examined by IF. As predicted, the level of M1 protein on WT cells was much higher than on mutant cells (Fig. 5C, D). Total M1 protein on any individual bacterial cell or chain of the Δ*dltA* mutant varied, but when immunoreactivity was observed it was mainly in the area between two bacteria in a chain. These inverse patterns of localization of M1 and C3 on WT and mutant cells provide evidence that restriction of antibody penetration does not play a role in either the flow cytometry or IF analysis.

**Figure 6:**
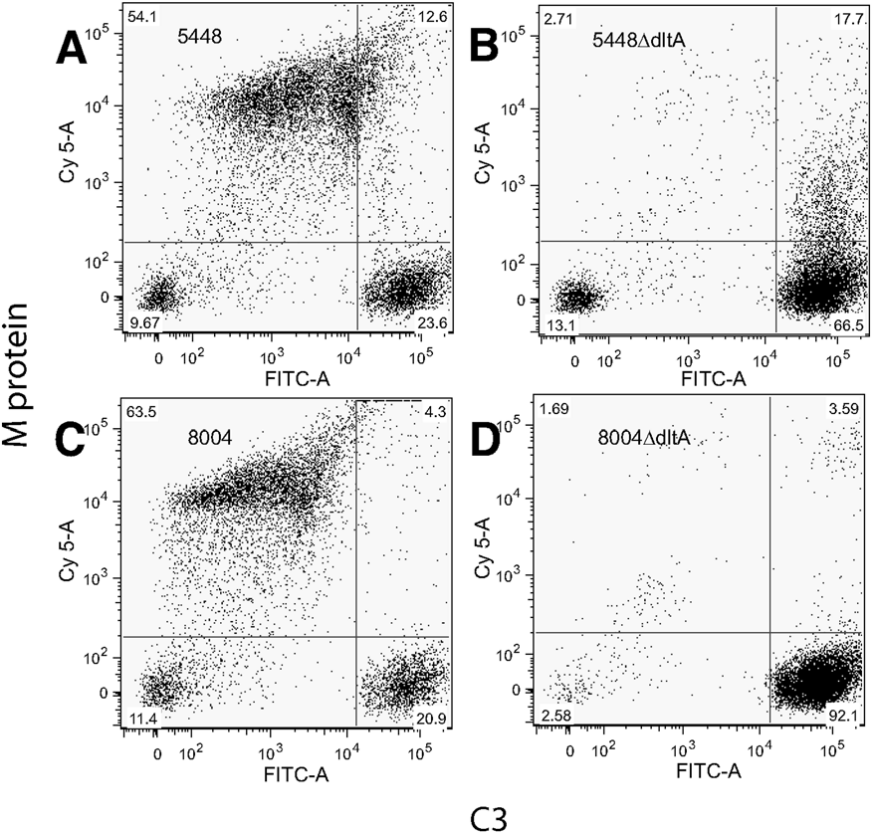
Comparing the relationship of complement deposition to M protein levels by flow cytometry. WT cells, 5448 (panel A) and 8004 (panel C), and their isogenic mutants, 5448Δ*dltA* (panel B) and 8004Δ*dltA* (panel D), were labeled with anti-C3 (FITC-A) and anti-M1 (Cy5-A). Numbers are given representing the % of cells in each gated area.

Inactivating the *emm* gene is known to result in high levels of complement binding in many serotypes, but relatively little is known about the direct correlation of complement binding as a function of the concentration of M protein expressed on the surface of individual cells. We used flow cytometry to further investigate the inverse relationship between C3 deposition and M1 expression. The flow cytometry profile shows that most of the mutant bacterial chains are both high in C3 deposition (FITC stain, Fig. 6B, D) and low in M1 protein levels (Cy5 stain, Fig. 6B, D). The WT cells showed a greater heterogeneity for both C3 deposition and M1 protein expression and a small (∼20%) subset of WT bacterial chains were low in M protein expression and high in complement binding (Fig. 6A, C, lower right quadrant). Chains exhibiting higher levels of M protein tended to have lower levels of C3 deposited on them (Fig. 6A, C, upper left quadrant).

Loss of LTA alanylation has been shown to result in rapid clearing of Δ*dltA* mutant bacteria from the blood, liver and spleen in an animal model [6]. We found that the growth of WT and Δ*dltA* mutant strains in blood (Table 2) was roughly correlated with surface M1 protein levels (see Fig. 2). Because there is inherent variability in these assays, it was performed using blood from two different donors, in three separate assays. In each case, the WT strain grew well, while the Δ*dltA* mutant was recovered in numbers of CFU that ranged from lower than were present in the inoculum to growth of only a few generations, suggesting that *in vivo* this strain could be efficiently cleared from the blood. In each case, the complemented strain, 5448Δ*dltA*(p*dltA*), showed increased resistance to phagocytosis, but did not return completely to WT levels.

**Table 2:** Growth of Bacteria in Human Blood.

Strain	Time point	Experiment		
		1^a^	2^b^	3^b^
5448				
	Inoculum CFU	290	166	211
	CFU after growth	4,950	88,250	81,666
5448 Δ*dltA*				
	Inoculum CFU	150	146	86
	CFU after growth	50	2,250	516
5448Δ*dltA*(p*dltA*)				
	Inoculum CFU	250	152	191
	CFU after growth	1,150	8,000	9,166

a Growth for 2 h at 37°C using blood of donor A.

b Growth for 2 h at 37°C using blood of donor B.

**Figure 7:**
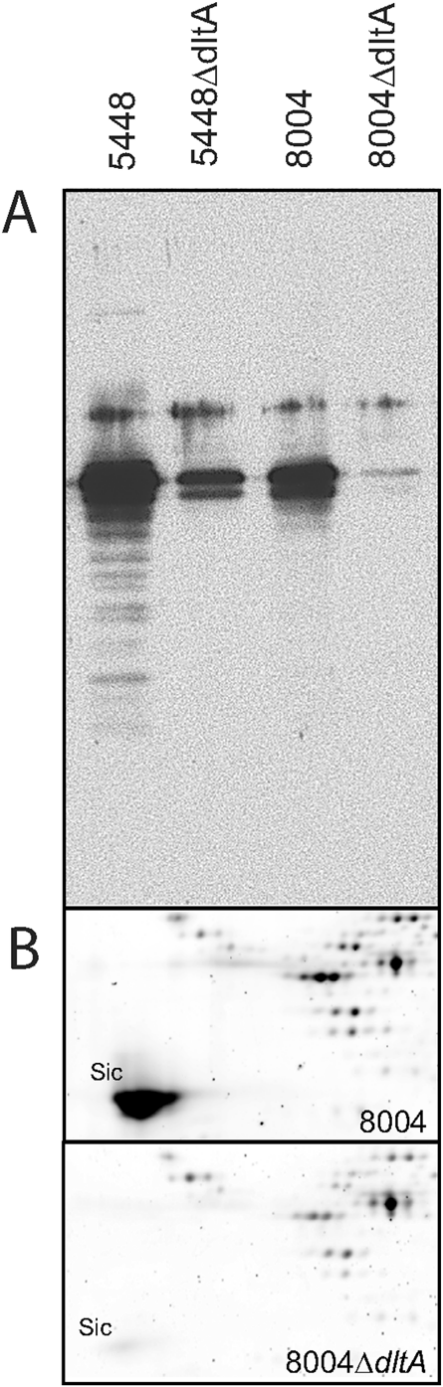
Analysis of the amounts of M protein and SIC released into the medium. Protein was precipitated from the supernatant of WT and *dltA* mutant bacteria grown to mid-log phase using TCA. A) Samples were separated by SDS-PAGE and blotted to nitrocellulose. Bands containing M1 protein were visualized using an anti-M1 antibody. B) Samples from 8004 and 8004Δ*dltA* were separated by 2-D gel electrophoresis. Only the portion of the gel surrounding SIC is shown. SIC was identified by mass spectrometry.

It is clear from the foregoing experiments that disruption of the *dltA* operon results in low levels of M1 surface protein, increased opsonization and phagocytosis, and marked loss of viability in whole human blood that can be at least partially complemented by restoring *dltA*. It was not clear from any of the *dlt* mutants previously described in the literature how the lack of D-alanylation of LTA might cause the expression of a surface protein to be lowered to such an extent. However, mutations in the *dlt* operon have been shown to result in perturbations at the cell membrane/cell wall interface and this has been linked to changes in post-transcriptional folding, degradation, and secretion of various proteins [8], [11], [14], [40]. To determine whether decreased M protein on the cell surface might be due to impaired retention, we examined M1 protein levels in the culture supernatant. We did not observe higher levels of M protein in the supernatant of mutants and in fact observed that the amount of M protein shed into the medium during log phase paralleled cell wall levels of M1 protein (Fig. 7A); there was much more M1 protein found in the supernatant from WT bacteria than from either Δ*dltA* mutant.

In addition to directly affecting protein function, the alteration of charge density at the cell membrane/cell wall interface could result in changes in the intracellular levels of ions or metabolites and/or changes in the cell's immediate environment, any of which could produce signals for regulatory circuit responses. To determine if the decrease in M protein was a result of transcriptional regulation, the amounts of *emm1* mRNA in WT and Δ*dltA* mutants during exponential growth were compared. Quantitative RT-PCR (qRT-PCR) measurements, normalized to the expression of gyrase (*gyr*), showed that *emm1* message was decreased by 50–200-fold in the *dltA* mutants in comparison with WT (Table 3). Although this does not exclude the possibility that M1 protein regulation might also occur at other levels, it is clear that the reduction of M1 protein in the *dltA* mutant was primarily due to a decrease in the level of *emm1* mRNA transcript. At the present time, we do not know whether the reduction in the level of transcripts is a result of decreased initiation of transcription or a decrease of message stability.

**Table 3:** Quantitative RT-PCR.

Strain	*emm*	*sic*	*mga*	*ccpA*	*rofA*	*rgg*	*arcA*
5448ΔdltA	0.020±0.020	0.054±0.054	0.193±0.003	1.100±0.011	0.870±0.004	19.503±0.282	2.045±0.101
8004ΔdltA	0.003±.003	0.004±0.004	1.683±0.134	1.646±0.295	0.890±0.134	5.686±0.393	6.6465±0.034

Relative Expression Levels*.

*WT transcript levels are set at 1.0.

Additional qRT-PCR measurements were performed in an effort to begin exploring possible mechanisms for decreased *emm1* expression. M protein expression is transcriptionally regulated by the stand-alone positive regulator, *mga* [19]–[22]. To determine if other members of the *mga* regulon were down-regulated in the mutants, we assayed the transcript levels of *sic* [41], [42] and found that it was similarly down-regulated (Table 3). Noting the reduction in SIC mRNA, we performed 2-D gel electrophoresis on culture supernatants of WT and Δ*dltA* mutants to determine whether SIC protein was also reduced in the mutant bacteria (Fig. 7B). SIC, which was identified by size, pI and mass spectrometry, was very much reduced in supernatants from 8004Δ*dltA* compared to those from WT 8004. The same degree of reduction of SIC was observed in supernatant from *S. pyogenes* 5448Δ*dltA* compared to 5448 (data not shown). Quantitation of *mga* mRNA by qRT-PCR demonstated the expected decrease in *mga* transcripts in the 5448Δ*dltA* strain. However, we were surprised to observe that the level of *mga* mRNA in 8004Δ*dltA*, which also bears a mutated *covS*, was essentially the same as the parent strain.

Transcription of the regulatory factor, Mga, is known to be positively regulated by itself [22] and by the carbon catabolite repression protein, CcpA (43), and negatively regulated by two other types of stand-alone transcription factors, RALPs (i.e. RofA and Nra) [44] and Rgg/RopB [44], [45]. qRT-PCR showed that although there were no significant differences in the levels of *ccpA* or *rofA* transcripts when WT and mutant bacteria are compared during exponential growth, the level of *rgg* transcripts was greatly increased in the Δ*dltA* mutants of both strains (Table 3). All of the data reported here were obtained using log-phase cells, which do not express the cysteine protease SpeB. However, since Rgg up-regulates SpeB and *rgg* was upregulated, we performed experiments comparing M protein levels in cells grown in the presence of the cysteine protease inhibitor, E64. There were no differences observed with or without the SpeB inhibitor (data not shown). The *arcA* gene encodes a member of the arginine deiminase system that converts arginine to ornithine, ammonia and CO_2_ to provide relief from acid stress. ArcA is another gene regulated by Rgg and it is also highly up-regulated in the Δ*dltA* mutants. Whether or not ArcA protein is expressed in greater amounts remains to be determined.

## Discussion

Previous studies have shown that inactivation of the *dlt* operon in a variety of Gram-positive bacteria results in pleiotropic phenotypes, including increased sensitivity to cationic AMPs and human group IIA phospholipase A2 [4], [12], [13], [27], [46], [47], decreased adhesion to epithelial cells [6], [13], [27], decreased ability to form biofilms [9], [27], increased autolysis [15], [14], [48], and increased acid sensitivity [7], [13]. In the present study, we demonstrated the effects of mutating *dltA* on virulence factor expression in *S. pyogenes* utilizing two distinct but closely related M1T1 isolates. The data reveal a novel role for *dltA* in the modification of the virulence phenotype of *S. pyogenes* through effects on M1 protein and SIC. These results should provide further impetus to the ongoing efforts [49] to target the D-alanylation pathway for potential therapeutic applications.

Our finding that the expression of M protein was greatly reduced in Δ*dltA* mutants of *S. pyogenes* compared to the parent strains is novel and somewhat unexpected, since a crude assessment of M protein levels by dot blot suggested that M protein levels were not grossly disparate between parent and mutant [13]. In the present study, a more detailed and comprehensive analysis was performed, and the reduction in expression of M1 protein was shown by several different techniques, including immunoblots of bacterial extracts, flow cytometry with whole bacteria, and qRT-PCR analysis of bacterial RNA. Several different procedures were used to extract streptococcal proteins. While there were quantitative differences in the amounts of protein extracted by the different procedures, the extracts of WT bacteria consistently contained much more M protein than did extracts of Δ*dltA* mutants. Differences in the rates of growth of the WT and Δ*dltA* mutants did not account for the differences in M protein contained in the extracts from each, as indicated by the essentially identical growth rates and confirmed by equivalent levels of total protein detected by SDS-PAGE. Failure to anchor M protein properly did not explain the differences, as confirmed by the greater amount of M protein in supernatants from WT than from Δ*dltA* bacteria. Differential digestion by the cysteine protease, SpeB, did not account for the differences, because all data were obtained from log-phase cells that do not express SpeB and because there was no effect of E64 on the outcomes observed. Therefore, we conclude that mutation of the Δ*dltA* gene, and by inference, the inability to esterify LTA with D-alanine, somehow affects the ability of *S. pyogenes* to express M protein.

The increased binding of C3 to the surface of *S. pyogenes* Δ*dltA* mutants, their increased interaction with PMN and their reduced ability to grow in whole human blood are all phenotypes associated with *S. pyogenes* that express suboptimal amounts of M protein. The surface M protein of *S. pyogenes* plays a major role in resistance to opsonization by complement in the non-immune host by binding host proteins that interfere with activation or binding of C3b [50]–[53]. The present study has shown, both by flow cytometry and IF, that the *dltA* mutation leads to an increase in C3b deposition concomitant with the reduction in M protein. As previously reported for other serotypes [54], [55], we observed that when *S. pyogenes* expressed WT levels of M protein on their surface, very little C3 was deposited, and its binding was restricted to small patches on the cell. C3 binding to the *dltA* mutants was very similar to that of an M-negative strain in which C3 is bound in greater amounts and deposited uniformly across the surface of the cell [54]. As expected, *dltA* mutant bacterial chains, which exhibited very high levels of C3 deposition, were more often associated with PMNs than were WT bacteria.

The expression of a secreted streptococcal virulence factor, SIC, is also reduced, as indicated by greatly reduced *sic* message and the virtual absence of SIC protein in culture supernatants. *Sic* is another member of the *mga* regulon in the M1 serotype [41]. SIC (and Drs, distantly related to Sic [42]) is expressed by only a few *S. pyogenes* serotypes, but it serves several critical roles in those isolates. Recombinant SIC protein has been shown to bind and inactivate several AMPs, including lysozyme, LL-37, and defensins [56], [57] and also to enter PMNs, bind to cytoskeletal proteins [58], and, thereby, to inhibit phagocytosis. DltA mutants in several bacterial species have been shown to have a greatly increased susceptibility to AMPs. This has been attributed to the increased binding of AMPs due to the increased negative charge that results from unesterified LTA, and it has been hypothesized that this may be the primary reason for the loss of virulence of *dltA* mutants *in vivo*. Our results indicate that the increased sensitivity of *S. pyogenes* M1T1 Δ*dltA* mutants to AMPs may also result from decreased expression of SIC, resulting in a reduced capacity to bind and inhibit AMPs at the bacterial cell surface. In addition, the significant reduction in the amount of M protein on the surface of the Δ*dltA* mutants significantly alters the binding of host proteins that could potentially block the binding and uptake of AMPs, thus rendering the mutants more susceptible to the antimicrobial action of these peptides.

Precisely how the inability to esterify LTA with D-alanine affects expression of M protein, SIC, or possibly other *S. pyogenes* virulence factors, is a key issue that remains to be investigated thoroughly. It seems clear that M protein and SIC are expressed at lower levels due to lowered expression of Mga. Most bacterial species use alterative sigma factors to coordinate widespread changes in their transcriptome as they transition through different phases of growth. *S. pyogenes*, however, relies on the interactions of the response regulator-controlled, growth-phase-oriented regulatory networks, including Mga, Nra/RofA, Rgg/RopB, and CovRS [21], [22], [44], [59]–[61]. These proteins are important for the appropriate expression of various virulence factor and metabolic genes at the proper stage of growth, as well as for the control of each other's expression. That *emm1* message is reduced in parallel with *mga* message in 5448Δ*dltA* is not surprising, but the data obtained thus far offer few, if any, clues as to how *mga* may be down-regulated in this strain. Rgg is known to suppress *mga* expression [61], [62] and *rgg* message is increased significantly in the Δ*dltA* mutant. The CovRS TCRS could be involved in both of these pathways, because it is known to up-regulate *rgg* expression and to suppress *mga* expression [61]. The best studied environmental signal for CovRS is Mg^2+^ [63] and LTA is known to bind Mg^2+^ [1], [5], [65]. LTA lacking D-alanine substitutions would be expected to bind even more Mg^2+^ [5], [65]. Furthermore, the net change in surface charge due to loss of D-ala could have a general effect on the ability of the bacteria to exchange of cations and anions across the cell wall, and this could affect one of the TCRSs or other regulatory pathways. However, these possibilities must be explored directly.

The potential link to CovRS is brought into question, however, when one considers the surprising qRT-PCR data from *S. pyogenes* 8004 and 8004Δ*dltA*. In the strain 8004 background, expression of both *emm* and *sic* were reduced in the Δ*dltA* strain, but expression of *mga* was either unchanged or was slightly elevated. This presents a conundrum not only regarding the potential CovRS link, but also regarding the link between *mga* and *emm*. Two possible explanations come to mind. One is that the transcription of *emm1* and *sic* is down-regulated by a Mga-independent mechanism in *S. pyogenes* 8004Δ*dltA*. This strain is a double mutant, bearing mutations in both *covS* and *dltA*, but there may be additional differences between 8004 and 5448. We expect that it is likely that there are feedback and/or compensatory mechanisms for gene regulation in *S. pyogenes* that remain to be identified. We found significant increases in the level of *rgg* transcripts in both *S. pyogenes* 5448Δ*dltA* and *S. pyogenes* 8004Δ*dltA* compared to WT. Both Mga and Rgg are stand-alone regulators, thus far without defined sensors. It is possible that repression of virulence gene expression in the two Δ*dltA* mutants is accomplished by two different regulatory pathways that share *rgg* as a common intersection in the altered regulatory network. Another possibility is that mRNA levels are regulated post-transcriptionally in 8004Δ*dltA*, perhaps at the level of mRNA stability. These interesting possibilities remain to be explored.

In summary, our findings indicate that ablation of *dltA* not only alters the ionic charge of LTA, but it also somehow modulates expression of at least two key virulence genes in two different isolates of serotype M1 *S. pyogenes*, the major serotype causing infections worldwide. Together with other published data on the effects of mutating the *dlt* operon, and the fact that the *dlt* operon is highly conserved among Gram-positive pathogens, our study adds greatly to the impetus for targeting the D-alanylation pathway in the search for novel antibacterial compounds.
